# FGFR3 drives Aβ-induced tau uptake

**DOI:** 10.1038/s12276-024-01274-3

**Published:** 2024-07-01

**Authors:** Dong Kyu Kim, Kyujin Suh, Junho Park, Sang-Eun Lee, Jihui Han, Sunghoe Chang, Youngsoo Kim, Inhee Mook-Jung

**Affiliations:** 1 Department of Biomedical Science, College of Medicine, Seoul National University, Seoul, Korea; 2 Convergence Research Center for Dementia, Medical Research Center, Seoul National University, Seoul, Korea; 3 Department of Medical Science, CHA University School of Medicine, Seongnam, Republic of Korea; 4 Advanced Omics Center, Future Medicine Research Institute, CHA Bundang Medical Center, Seongnam, Republic of Korea; 5 Neuroscience Research Institute, Seoul National University College of Medicine, Seoul, South Korea

**Keywords:** Alzheimer's disease, Molecular neuroscience

## Abstract

The amyloid cascade hypothesis suggests that amyloid beta (Aβ) contributes to initiating subsequent tau pathology in Alzheimer’s disease (AD). However, the underlying mechanisms through which Aβ contributes to tau uptake and propagation remain poorly understood. Here, we show that preexisting amyloid pathology accelerates the uptake of extracellular tau into neurons. Using quantitative proteomic analysis of endocytic vesicles, we reveal that Aβ induces the internalization of fibroblast growth factor receptor 3 (FGFR3). Extracellular tau binds to the extracellular domain of FGFR3 and is internalized by the FGFR3 ligand, fibroblast growth factor 2 (FGF2). Aβ accelerates FGF2 secretion from neurons, thereby inducing the internalization of tau-attached FGFR3. Knockdown of FGFR3 in the hippocampus reduces tau aggregation by decreasing tau uptake and improving memory function in AD model mice. These data suggest FGFR3 in neurons as a novel tau receptor and a key mediator of Aβ-induced tau uptake in AD.

## FGFR3 Identified as Key Tau Receptor in Alzheimer’s Progression

Alzheimer’s disease (AD), the most common dementia leading to progressive memory loss and cognitive decline, is characterized by the accumulation of two pathological proteins in the brain: amyloid beta(Aβ) and tau. A recent study found that Aβ accelerates tau pathology in the brain, worsening AD. Using mice models, research showed that fibroblast growth factor receptor 3 (FGFR3) can act as tau receptor, and Aβ increases the tau uptake and aggregation by FGFR3 in brain cells. The findings suggest that decreasing FGFR3 could significantly lessen tau toxicity in brain cells. This could provide a new approach to slow down AD progression by targeting the early stages of tau accumulation. The study paves the way for potential treatments that could delay or prevent AD progression by targeting the early interaction between Aβ and tau. This summary was initially drafted using artificial intelligence, then revised and fact-checked by the author.

## Introduction

Alzheimer’s disease (AD) is clinically characterized by memory impairment and is pathologically characterized by the accumulation of extracellular amyloid beta (Aβ) plaques and neurofibrillary tangles (NFTs) composed of tau aggregates in the brain^1^. AD biomarkers have been developed to predict clinical progression, and AD-associated brain pathology has been found to proceed in a specific temporal sequence with disease progression^2^. Increased concentrations of Aβ in the cerebrospinal fluid (CSF) and Aβ deposition in the brain are observed before the onset of other AD symptoms; these changes are followed by hippocampal atrophy, increased concentrations of tau in the CSF, and ultimately, cognitive impairment^3,4^. The amyloid hypothesis, which suggests that Aβ aggravates tau pathologies and promotes tau propagation from the entorhinal cortex to the hippocampus, is supported by multiple experimental models and animal models of AD^5–7^.

The Aβ pathology that appears first in the AD brain can lead to many cellular and molecular changes, supporting the amyloid cascade hypothesis for AD progression^8^. Aβ plays a crucial role as the initiating factor for disease occurrence, such as by activating glia and altering kinase/phosphatase activities, leading to NFT formation^9,10^. Previous research on the pathogenic interaction between Aβ and tau has focused on the ability of Aβ to act as an activator of tau aggregates, promote posttranslational modification of tau, or inhibit tau degradation in neurons^7,11–15^. A recent study proposed a remote regional Aβ–tau interaction in which Aβ preferentially determines the direction of tau spreading, explaining the topographical dissimilarity between early Aβ and tau depositions^16^. Glial activation has recently been highlighted as a critical intermediary for Aβ–tau synergy to elucidate the remote interaction model. The Aβ-mediated release of proinflammatory cytokines from activated microglia enhances tau aggregation^17,18^. A recent study using positron emission tomography imaging of the brain supported the amyloid cascade hypothesis by revealing associations between microglial activation, tau pathologies, and Aβ depositions in AD brains^19^. Correlation analysis revealed that Aβ deposition induces microglial activation to affect tau propagation (assessed according to Braak stages)^19^. Other evidence suggests that Aβ deposition and tau accumulation synergistically affect AD progression through pathogenic interactions^20^.

We studied whether preexisting Aβ synergistically influences tau absorption in neurons, one of several phases in which Aβ may directly influence tau propagation. We show here that preexisting Aβ accelerates the internalization of extracellular tau. A proteomic study of endocytic vesicles revealed that Aβ-dependent fibroblast growth factor receptor 3 (FGFR3) regulates extracellular tau internalization. The interaction between FGFR3 and extracellular tau promotes tau absorption and subsequent tau pathologies and is regulated by the FGFR3 ligand, fibroblast growth factor 2 (FGF2). Our findings show that FGFR3 is an important modulator of tau uptake and Aβ–tau synergy in AD, suggesting that modulating the FGF2–FGFR3 pathway could slow the progression of tau pathology in AD.

## Materials and methods

The methods are described in detail in the Supplementary material.

### Animals

5XFAD (Tg6799; Jackson Laboratory, stock #006554) mice^21^, which carry three mutations (K670N/M671L, Swedish; I716V, Florida; V717I, London) of human amyloid precursor protein (APP) and two mutations (M146L and L286V) of human presenilin-1 (PS1) driven by the neuron-specific promoter (Thy-1), were used for the assessment of FGF2 and FGFR3. For AAV-siFGFR3 virus injection experiments, ADLP^APT^ model mice generated by crossing 5XFAD mice and JNPL3 mice (TauP301L-JNPL3; Taconic, Stock#2508 homozygous), which exhibit AD-like pathology, amyloid lesions and tauopathy, were used^6^. For the brain extraction of tau transgenic mice, ADLP^Tau^ mice, which were generated by crossing 5XFAD mice and JNPL3 mice and exhibited only tauopathy, were used. Female mice were used for all experiments. Animals were treated and maintained following the Animal Care and Use Guidelines of Seoul National University, Seoul, Korea.

### Stereotaxic injection

Mice were anesthetized with a mixture of Zoletil 50 (Virbac) and Rompun (Bayer). AAV solution or preformed tau fibrils (PFFs, Stressmarq) were stereotaxically injected into the hippocampus with a Hamilton needle (0.2 μl/min; AP, -2.0 mm; ML, +1.3 mm; DV, +2.0 mm from the bregma). The syringe was removed from the brain 10 min after the infusion. Virus-injected mice were maintained for 12 weeks postinjection before being used in experiments. After injections of PFFs, the mice were maintained for 4 weeks before performing the immunohistochemical experiments.

### Behavior tests

#### Y-maze test

At 12 weeks post virus injection, the mice were placed in a Y-maze and allowed to explore freely for 8 min. We traced and counted the entries into each arm of the maze. Spontaneous alternation and the total number of arm entries were recorded. To calculate the percentage of spontaneous alternation, the number of entering three different arms in a row was divided by the number of total arm entries. The total number of entries was used to evaluate the mobility and activity of the mice.

#### Contextual fear conditioning (CFC) test

On Day 1, mice were placed for a total of 270 s in an isolation cubicle with an electrifiable grid floor (Coulbourn); at 180 and 240 s, they were given 0.55 mA foot shocks for 2 s each. On Day 2, the protocol described for Day 1 was repeated without any foot shock. Each mouse was allowed to freely explore the same chamber for 5 min, and freezing behavior was analyzed by FreezeFrame software (Coulbourn).

#### Novel object recognition test

On Day 1, the mice were placed in an opaque Plexiglas square chamber (40 cm×40 cm) for 10 min to allow free exploration. On Day 2, the mice were placed in the same chamber to explore for 10 min, with two identical objects placed at equal distances in the arena. On Day 3, one object was replaced with a novel object of a similar size, and the mice were placed in the chamber for 5 min while being recorded. The object-exploring time was measured as the time needed for the subject to touch its nose to the object or orientation toward the object within 1 cm. The discrimination index was calculated as (time spent exploring the novel object)/(time spent exploring the familiar object) + (time spent exploring the novel object).

### Primary neuron culture

All embryos from ICR mice at E16–17 were obtained, and their cerebral cortices were isolated after removing the meninges from the brain. The minced cortices were incubated in HBSS with 0.05% trypsin at 37 °C for 30 min. Then, a trypsin inhibitor was added to stop the digestion, and the tissues were transferred to neurobasal media (NB, neurobasal medium supplemented with 10% FBS and 1% P/S) supplemented with DNase. After further dissociating the tissues by pipetting, single cells were collected from the supernatant. The cells were resuspended in fresh NB media, counted, and seeded in 6- or 12-well plates coated with PDL at 78 k/cm^2^. New medium was added to the plate every 2-3 days. At DIV14, primary cortical neurons were treated with 3 μM Aβ in NB medium. After 3 days of incubation, the neuronal media was fully removed, and the neurons were incubated in fresh NB media mixed with 10 nM human tau (wild-type or P301L mutant) for 4 days. At DIV21, the neurons were used in biochemical experiments.

### Preparation of Aβ

The preparation of synthetic Aβ and oligomerization were performed as previously described^22^. Aβ_1–42_ peptides (Bachem) were dissolved in 1,1,1,3,3,3-hexafluoro-2-propanol (HFIP; Sigma‒Aldrich) to a final concentration of 1 mM. HFIP was evaporated under vacuum using a SpeedVac Concentrator (Savant Instruments), and the dried peptides were stored at -80 °C. Monomeric Aβ was prepared by dissolving Aβ_1–42_ peptides at 1 mM in anhydrous dimethyl sulfoxide (anhydrous DMSO; Sigma‒Aldrich) and used immediately. Oligomeric Aβ was prepared by diluting the monomeric Aβ solution to 10 μM in PBS and incubating the solution overnight at 4 °C.

### Brain extracts of tau transgenic mice

We extracted the brains of tau transgenic mice using a modified protocol as described previously^23^. Tau transgenic mice (16-month-old ADLP^Tau^ mice), which overexpress human mutant P301L tau, were perfused with PBS, and their brains were isolated and frozen in liquid nitrogen. Brain tissue was homogenized with five volumes (wt/vol) of PBS containing a cocktail of protease and phosphatase inhibitors using a pestle mixer. The brain homogenate was sonicated and centrifuged at 3,000 × g for 5 min at 4 °C. The supernatant was collected and centrifuged at 100,000 × g for 30 min at 4 °C to make the pellet, which was then stored at -80 °C before use.

### Western blotting

Cells or tissues were lysed in RIPA buffer containing a cocktail of protease and phosphatase inhibitors. Lysates of protein (10 μg) were separated on NuPAGE 4–12% Bis-Tris gels (Thermo Fisher Scientific) in MES buffer (Thermo Fisher Scientific) and transferred to PVDF membranes. The membranes were blocked with 5% skim milk in TBST (Tris-buffered saline containing 0.1% Tween 20) for 1 hr, incubated overnight with primary antibodies at 4 °C, and incubated with secondary antibodies for 1 hr at room temperature. The protein bands were visualized by enhanced chemiluminescence (ECL) western blotting detection reagents (Ab Frontier) and detected using an LAS-3000 image analyzer (Fuji Photo Film).

### Tau fractionation

Tau fractionation was performed as previously described^6^. In brief, one side of the hippocampus was homogenized in Tris-buffered saline (TBS; 25 mM Tris–HCl, pH 7.4; 150 mM NaCl; 1 mM EDTA; and 1 mM EGTA with protease and phosphatase inhibitors). The tissue homogenate was centrifuged at 14,000 × g at 4 °C for 15 min. The resulting supernatant was incubated with 20% N-Lauroylsarcosine sodium salt solution (final 1%; L7414, Sigma‒Aldrich) at 37 °C for 1 hr. The mixture was ultracentrifuged at 150,000 × g for 45 min at 25 °C. The supernatant was collected as a sarkosyl-soluble fraction, and the pellet was washed with 1% sarkosyl solution in TBS and ultracentrifuged again at 150,000 × g for 1 hr at 25 °C. The resulting pellet was collected as a sarkosyl-insoluble fraction. All fractions were boiled with sample buffer (Serva blue G) at 70 °C for 10 min.

### Immunocytochemistry

HT22 cells were plated on PDL-coated glass slides, washed with PBS, and fixed in 4% paraformaldehyde for 20 min at room temperature. The fixed cells were permeabilized with PBS containing 0.1% Triton X-100 and 10% normal horse serum for 1 hr at room temperature and then incubated with primary antibodies overnight at 4 °C. The primary antibodies used included anti-Tau13 (ab19030, Abcam; 1:500 or #835201, Biolegend, 1:200), anti-FGFR3(PA5-34574, Thermo, 1:200), anti-CHMP2B (ab33174, Abcam; 1:100), anti-LAMP1 (ab25245, Abcam, 1:200), anti-LC3B (NB600-1384, Novus Biologicals, 1:200), and anti-MAP2 (ab5392, Abcam, 1:1000) antibodies. The cells were incubated with Alexa Fluor 488- or 594-conjugated anti-rabbit or anti-mouse IgG for 1 hr, and the cell nuclei were stained with DAPI. The cells were imaged using a confocal laser microscope (Zeiss) with 40× and 63× water-immersion objective lenses, and the images were processed using ZEN software (Zeiss).

### Extracellular vesicle isolation

We purified extracellular vesicles (EVs) from tau-conditioned medium using a modified protocol as described previously^24^. The tau-conditioned medium was centrifuged at 1,200 × g for 20 min at 4 °C to remove cell debris, the supernatant was filtered through a 0.22 μm syringe filter (Merck Millipore) to remove apoptotic bodies larger than EVs, and then the filtered sample was ultracentrifuged at 100,000 × g for 70 min at 4 °C. The supernatant was collected as non-EV medium, and the EV pellet was suspended in PBS.

### Endocytic vesicle fractionation

Endocytic vesicles were isolated as described previously^25^. Adherent HT22 cells treated with tau-conditioned medium were washed with PBS. Cells were scraped in 10 ml of MES buffer (0.1 M 2-(*N*-morpholino) ethanesulfonic acid [MES], pH 6.5 [adjusted with NaOH], 0.2 mM ethylene glycol tetraacetic acid, and 0.5 mM MgCl_2_) and lysed with a 30 ml glass Dounce homogenizer, and the cell lysate was centrifuged at 4000 × g for 30 min at 4 °C. The resulting pellet containing unbroken cells was used as a total sample. The supernatant was incubated with RNaseA (50 μg/ml) for 1 hr at 4 °C and centrifuged at 4000 × g for 5 min, and the supernatant obtained from that step was ultracentrifuged at 209,000 × g for 50 min at 4 °C. The obtained membrane pellet was washed with 400 μl of MES buffer and lysed with a 1 ml Wheaton homogenizer (tight pestle). An equal volume of F/S buffer (12.5% [wt/vol] Ficoll and 12.5% [wt/vol] sucrose in MES buffer) was added, and the mixture was centrifuged at 21,800 × g for 40 min at 4 °C. Finally, the resulting supernatant was mixed with MES buffer (1:5 ratio) and centrifuged at 195,500 × g for 50 min at 4 °C to obtain the endocytic vesicle pellet.

### Proteomics experiment

Endocytic vesicles enriched from HT22 recipient cells were lysed and isolated by acetone precipitation, as previously described^6,26^. Protein digestion was carried out using a filter-aided sample preparation (FASP) approach, as previously described^6,26,27^. The obtained peptides were acidified with 10% trifluoroacetic acid (TFA) and desalted using custom C18 StageTips, as previously described^28^. Prior to MS analysis, the lyophilized peptides were dissolved in solvent A (2% ACN and 0.1% formic acid, v/v) and separated by reversed-phase chromatography using an Easy-nano LC 1000 (Thermo Fisher Scientific) instrument. The peptides were separated using a 2 column setup with a trap column (Thermo Fisher Scientific; 75 μm I.D. × 2 cm long, 3 μm Acclaim PepMap100 C18 beads) and an analytic column (Thermo Fisher Scientific; 75 μm I.D. × 15 cm long, 3 μm ReproSil-Pur-AQ C18 beads). The separated peptides were electrosprayed by an electrospray ionization source at an ion spray voltage of 2200 eV. MS spectra were acquired in a data-dependent manner using a top-15 method on a Q Exactive Orbitrap mass spectrometer (Thermo Fisher Scientific). MaxQuant software (version 2.1.4.0) was used to perform a database search, as described previously^29,30^. The confidence criterion was a 1% false discovery rate (FDR) at the peptide and protein levels. Proteins with an iBAQ intensity in at least 70% of the samples in each group were subjected to further statistical analysis. The statistical analysis was carried out using Perseus software (ver. 1.6.15.0)^31^, and functional classification was carried out using DAVID bioinformatics^32^.

### Total internal reflection fluorescence (TIRF) microscopy

HT22 cells were immunostained with anti-FGFR3 and anti-Tau13 antibodies following the immunocytochemistry protocol. Images were acquired with an N-STORM (Nikon) microscope equipped with a 100X TIRF objective lens (1.4 NA) and an iXon3 897 EMCCD (Andor Technologies, Belfast, Northern Ireland). The microscope was focused on the adherent plasma membrane, and images were acquired in either epifluorescence or TIRF mode.

### Statistical analysis

The data are presented as the mean ± standard error of the mean (SEM). The statistical analyses performed included unpaired t tests, one-way analysis of variance (ANOVA), and two-way ANOVA followed by Tukey’s test for multiple comparisons. The accepted levels of significance were *P < 0.05, **P < 0.01, ***P < 0.001, and ****P < 0.0001. Statistical analysis was performed using the Prism 8 software package (GraphPad Software, USA) for Windows.

## Results

### Aβ increases extracellular tau uptake

**Fig. 1:**
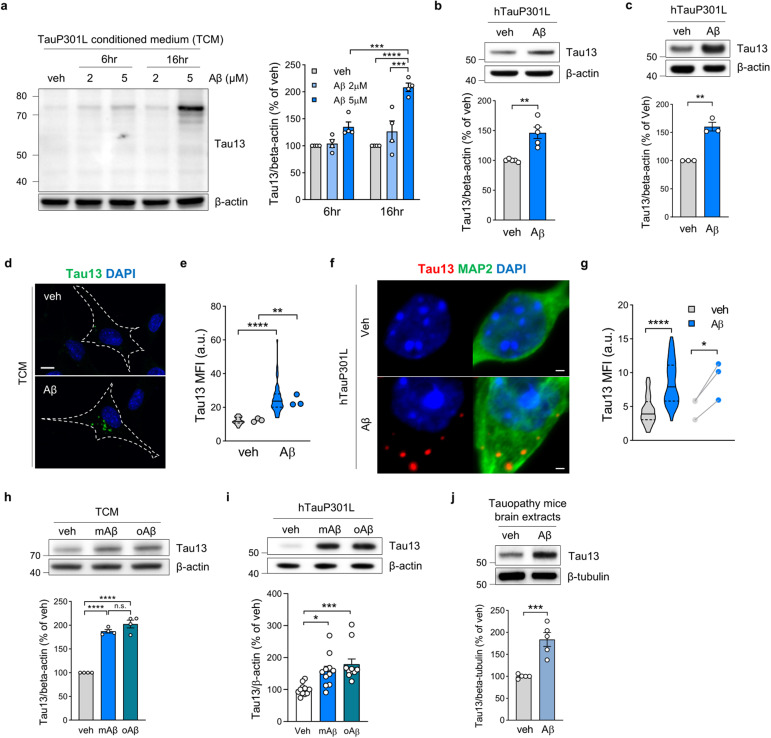
Aβ accelerates extracellular tau uptake. **a** Representative immunoblots and quantification of human tau in recipient cells. Human tau signals were normalized to those of β-actin. Two-way ANOVA. **b**, **c** Representative immunoblots and quantification of human tau levels in HT22 cells (**b**) and primary neurons (**c**) following synthetic tau treatment. Unpaired two-tailed t test. Representative image (**d**) and quantification (**e**) of internalized human tau in HT22 cells. Unpaired two-tailed t test (*n* = 39 cells, 3 replicates/group). Scale bar, 10 μm. Representative image (**f**) and quantification (**g**) of internalized human tau in primary neurons following synthetic tau treatment. Paired two-tailed t test (*n* = 49-55 cells, 3 replicates/group). Scale bar, 1 μm. **h**, **i** Representative immunoblots and quantification of human tau in HT22 cells (**h**) and primary neurons (**i**) after pretreatment with different Aβ forms (5 μM monomeric, mAβ or oligomeric Aβ, oAβ). Human tau signals were normalized to those of β-actin. One-way ANOVA with Tukey’s test. **j** Representative immunoblots and quantification of human tau in primary neurons following treatment with brain extracts from tauopathy model mice. Unpaired two-tailed t test. The data are presented as the mean ± SEM. **P < 0.01, ***P < 0.001, and ****P < 0.0001.

To determine whether Aβ affects tau internalization, we transferred tau-conditioned medium (TCM), a source of extracellular human tau P301L, to Aβ-pretreated HT22 mouse hippocampal neurons. We found that the internalization of full-length extracellular tau by HT22 cells depended on pretreatment with Aβ in a time- and dose-dependent manner (Fig. 1a). We also used synthetic human tau as a different source of extracellular tau to examine the translatability of Aβ-induced tau uptake in HT22 cells and primary neurons. We found that Aβ increased the uptake of synthetic human tau by HT22 cells and primary neurons (Fig. 1b, c). Through immunocytochemistry, internalized tau in punctate structures was detected in the cytosol of HT22 cells (Fig. 1d, e) and in both neurites and soma of primary neurons (Fig. 1f, g, Supplementary Fig. 1). Oligomeric Aβ, as well as monomeric Aβ, also increased internalized tau levels in HT22 cells (Fig. 1h) and in primary neurons (Fig. 1i). Brain extracts of tau transgenic mice containing a rare phosphorylated high-molecular-weight tau are taken up by neurons and are involved in tau propagation^23^. In primary neurons pretreated with Aβ, tau uptake was observed in brain extracts from tau transgenic mice (Fig. 1j). Tau internalization accelerated by Aβ also occurred with TCM with wild-type human tau and synthetic wild-type human tau (Supplementary Fig. 2).

Tau is released through various mechanisms to cause cell-to-cell tau propagation^33,34^. Extracellular tau can be found in multiple forms, including in its free form or within membrane vesicles^35,36^, and it is secreted via diffusion or membranous organelles, such as autophagic vesicles, exosomes, and ectosomes^37,38^. To determine the form of tau in TCM, we fractioned TCM into extracellular vesicle (EV) and non-EV fractions and measured their tau levels. Most extracellular tau in TCM was found in the non-EV fraction containing a free form of secreted tau (Supplementary Fig. 3a, b). The free form of human tau in the non-EV fraction was predominantly internalized by Aβ pretreatment (Supplementary Fig. 3c).

Aβ may engage in two aspects of cell-to-cell tau spreading: secretion and uptake^39,40^. Neuronal hyperactivity enhances tau secretion, as shown by optogenetic neuronal modulation in tauopathy model mice^41^. To determine whether Aβ affects tau secretion from neurons, we measured secreted tau levels in TCM after Aβ treatment. Tau secretion was not induced by Aβ, as assessed by western blot and human tau ELISA (Supplementary Fig. 4a, b), nor was it induced by Aβ treatment of primary neurons expressing human tau (Supplementary Fig. 4c). These findings indicate that Aβ contributes to neuronal tau uptake but not to tau secretion.

### Aβ drives extracellular tau into endocytic vesicles

**Fig. 2:**
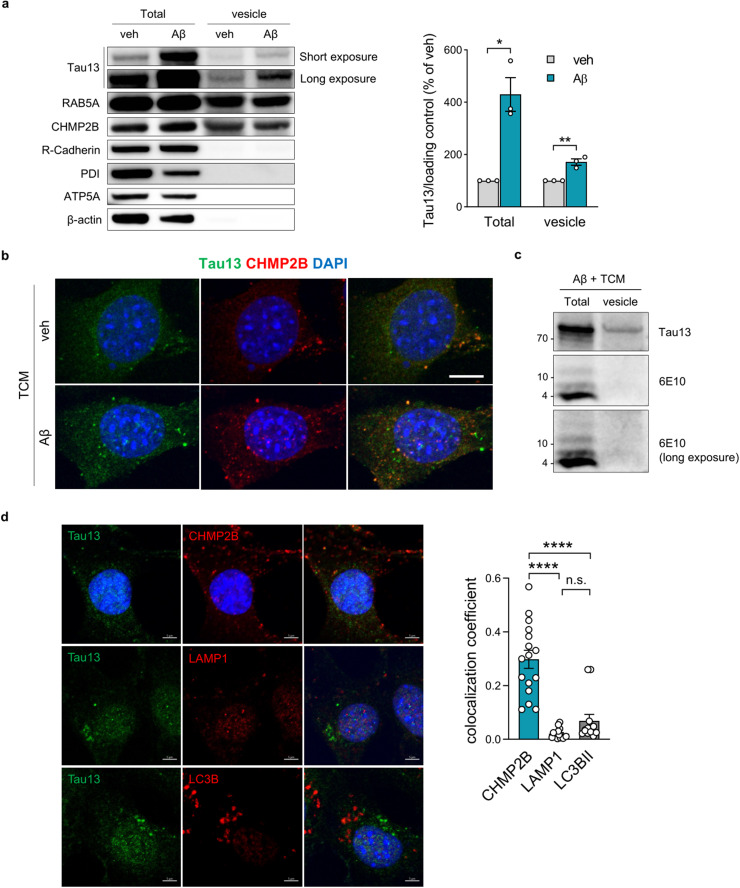
Internalized tau induced by Aβ is localized in endocytic vesicles. **a** Representative blots and quantification of internalized tau in endocytic vesicles isolated from HT22 cells pretreated with Aβ (5 μM) followed by incubation in TCM. The fractioned vesicles were verified with subcellular markers of endocytic vesicles (RAB5A), multivesicular bodies (CHMP2B), the plasma membrane (R-cadherin), the endoplasmic reticulum (PDI), and mitochondria (ATP5A). Human tau signals were normalized to those of β-actin for total cell lysates and to those of CHMP2B for fractioned endocytic vesicles. Unpaired two-tailed t test. **b** Immunocytochemistry of internalized human tau colocalized with the MVB marker CHMP2B. Scale bar, 10 μm. **c** Western blot analysis of total cell lysates and endocytic vesicles showing the presence of tau and the absence of Aβ in fractioned endocytic vesicles. **d** Representative images and the colocalization coefficient between internalized tau and each subcellular organelle in HT22 recipient cells pretreated with Aβ. Scale bar, 5μm. One-way ANOVA. The data are presented as the mean ± SEM. *P < 0.05, **P < 0.01, and ****P < 0.0001.

To trace the movement of extracellular tau in the presence of Aβ, we analyzed tau levels in endocytic vesicles isolated from recipient cells. The fractionation was verified by the presence of marker proteins for early endosomes (RAB5A) and late endosomes/multivesicular bodies (CHMP2B) and the absence of markers for the plasma membrane (R-cadherin), mitochondria (ATP5A), or the endoplasmic reticulum (PDI). The endocytic vesicles contained extracellular tau, and the amount of tau was increased by pretreatment with Aβ (Fig. 2a). These results were validated by immunostaining for internalized tau, which demonstrated that internalized tau formed punctate structures that colocalized with CHMP2B (Fig. 2b). In contrast, Aβ was not detected in endocytic vesicles, indicating that pretreatment with Aβ did not induce tau uptake through physical interaction with tau (Fig. 2c).

To identify where internalized tau is localized in the endolysosomal pathway, we analyzed the colocalization coefficient between internalized tau and each compartment marker of the endolysosomal pathway, including CHMP2B, LAMP1, and LC3B. The colocalization coefficient between internalized tau and CHMP2B was significantly greater than that between internalized tau and other markers, suggesting that internalized tau is mostly localized in the early endosome, the CHMP2B-positive compartment, rather than in the late endolysosomal pathway (LC3B or LAMP1) (Fig. 2d). Therefore, our data suggest that Aβ drives extracellular tau into endocytic vesicles but not by directly interacting with tau.

### Aβ induces tau uptake via FGFR3

**Fig. 3:**
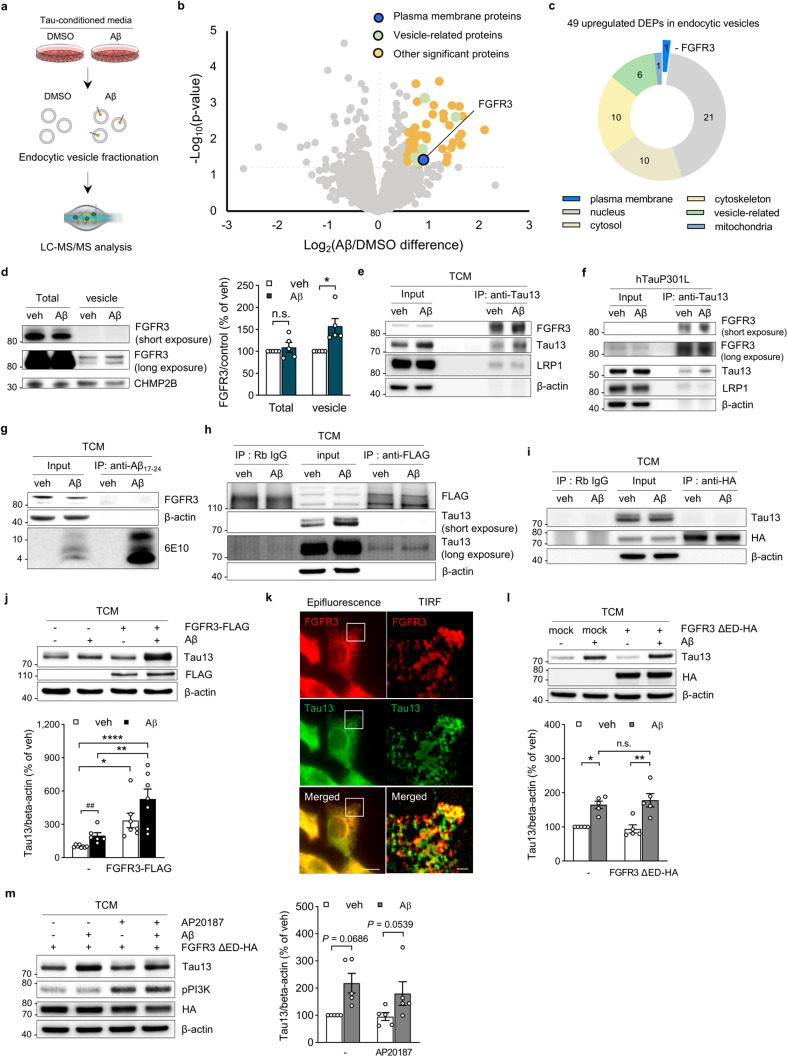
FGFR3 is a key mediator of Aβ-induced tau uptake. **a** Schematic overview of the proteomic analysis of endocytic vesicles used to identify differentially expressed proteins (DEPs) by Aβ. **b** Volcano plot of DEPs in endocytic vesicles after Aβ or DMSO treatment. **c** Subcellular localization of 49 upregulated DEPs (p value < 0.05). **d** Representative immunoblots and quantification of FGFR3 in total lysates and endocytic vesicles in response to Aβ. FGFR3 levels were normalized to those of β-actin for the total lysate and CHMP2B for the vesicle sample. Unpaired two-tailed t test. **e** Immunoblots of coimmunoprecipitation of human tau in HT22 cells. **f** Immunoblots of coimmunoprecipitation with human tau in primary neurons. **g** Immunoblots of HT22 cells coimmunoprecipitated with Aβ. **h** Immunoblots of coimmunoprecipitation of FGFR3 in HT22 cells overexpressing FGFR3-FLAG. **i** Immunoblots of coimmunoprecipitation with the extracellular domain deletion mutant FGFR3 in HT22 cells overexpressing FGFR3 ΔED-FKBP12v36-HA. **j** Representative immunoblots and quantification of human tau in HT22 cells overexpressing FGFR3-FLAG. Human tau signals were normalized to those of β-actin. Two-way ANOVA. **k** TIRF images of extracellular tau binding to FGFR3 on the cell membrane. Representative epifluorescence and enlarged TIRF images showing the interaction of FGFR3 (red) and tau (green) in HT22 cells following Aβ pretreatment. Scale bars, 10 µm (top) and 2 µm (bottom). **l** Representative immunoblots and quantification of human tau in HT22 cells overexpressing the FGFR3 extracellular domain deletion mutant FGFR3 ΔED-FKBP12v36-HA. Human tau signals were normalized to those of β-actin. Two-way ANOVA. **m** Representative immunoblots and quantification of human tau in HT22 cells overexpressing FGFR3 ΔED-FKBP12v36-HA dimerized with 5 nM AP20187 (FKBP dimerizer). Human tau signals were normalized to those of β-actin. Two-way ANOVA. The data are presented as the mean ± SEM. *P < 0.05, **P < 0.01, and ****P < 0.0001.

To explore the mechanism by which Aβ increases tau uptake, we investigated quantitative proteomic differences in endocytic vesicles isolated from recipient HT22 cells treated with DMSO or Aβ using label-free quantification (Fig. 3a). We identified 49 differentially expressed proteins (DEPs) in endocytic vesicles between the DMSO- and Aβ-treated groups (Supplementary Table. 1). Among these DEPs, FGFR3 was the only plasma membrane-localized protein (Fig. 3b, c). These results demonstrate that FGFR3 internalization by Aβ may coincide with Aβ-induced tau uptake, suggesting an association between the two phenomena. Thus, we further validated the internalization of FGFR3 by Aβ and investigated how this affects Aβ-induced tau uptake.

FGFR3 levels were examined in both total cell lysates and endocytic vesicles of recipient cells by western blotting. Compared with those in the control group, the FGFR3 levels in endocytic vesicles were greater in the Aβ-treated group, whereas the FGFR3 levels in total lysates were not altered by the presence of Aβ (Fig. 3d). These results demonstrate that Aβ induces the internalization of FGFR3 without affecting its expression, which led us to investigate whether this effect might be related to the mechanism through which Aβ induces tau uptake.

We next tested whether FGFR3 mediates Aβ-induced tau uptake via direct interaction with tau. Tau in the recipient cell lysates was immunoprecipitated with an anti-human tau antibody (Tau13). In HT22 cells and primary neurons, we found that extracellular tau binds to FGFR3 as well as to LRP1, which was recently reported as a tau receptor in neurons (Fig. 3e, f, and Supplementary Fig. 5)^42^. Interestingly, FGFR3 played a distinct role in Aβ-induced tau uptake and showed a high level of binding to tau despite its relatively low expression levels in neurons. Immunoprecipitation of Aβ with an anti-Aβ_17-24_ antibody revealed that FGFR3 did not coprecipitate with Aβ (Fig. 3g), indicating that preexisting Aβ did not directly interact with FGFR3 to induce the internalization of FGFR3 and tau.

To further support these findings, we immunoprecipitated FGFR3 from HT22 cells expressing FGFR3-FLAG with an anti-FLAG antibody. Consistent with the results described above, we found that FGFR3-FLAG bound to extracellular tau (Fig. 3h). To test the hypothesis that tau binds to the extracellular domain of FGFR3 and thereby undergoes internalization, we tested the interaction of tau with the cytosolic domain of FGFR3 in HT22 cells expressing the FGFR3 extracellular domain deletion mutant (FGFR3 ΔED-FKBP12v36-HA). Immunoprecipitation of the FGFR3 extracellular domain deletion mutant with an anti-HA antibody showed no coimmunoprecipitation of tau, indicating that extracellular tau binds to the extracellular domain of FGFR3 (Fig. 3i).

To further investigate the role of FGFR3 in Aβ-induced tau uptake, we observed tau uptake in HT22 cells overexpressing either full-length FGFR3 or FGFR3 extracellular domain deletion mutants. HT22 cells overexpressing full-length FGFR3 showed significantly increased levels of internalized tau when recipient cells were pretreated with Aβ (Fig. 3j). Additionally, to examine the interaction between extracellular tau and FGFR3 on the cell surface, an optical section of the recipient cell surface was imaged by total internal reflection microscopy (TIRF). On the surface of the recipient cells, extracellular tau colocalized with FGFR3. As the initial step of the tau uptake process, extracellular tau first bound to FGFR3 on the surface of recipient cells (Fig. 3k). However, HT22 cells overexpressing the FGFR3 extracellular domain deletion mutant did not show an increase in tau uptake (Fig. 3l). These data are consistent with our immunoprecipitation findings showing that the FGFR3 extracellular domain is required for tau uptake (Fig. 3h, i). Upon the binding of FGF, FGFR3 dimerizes and autophosphorylates on its cytosolic tyrosine kinase domain, enabling it to act in further signaling pathways^43^. To determine whether downstream signaling of FGFR3 could influence tau uptake, we chemically induced dimerization of the FGFR3 cytosolic domain in HT22 cells overexpressing FGFR3 ΔED-FKBP12v36-HA. AP20187 was used to cause dimerization of the FKBP domain with the F36V mutant FGFR3, which directs constitutive activation of the downstream signaling cascade^44^. AP20187-induced dimerization of the FGFR3 cytosolic domain and activation of downstream signaling did not affect tau uptake (Fig. 3m). Thus, tau uptake depends on the physical interaction between FGFR3 and extracellular tau, not on the downstream signaling pathway of FGFR3. Together, these results suggest that FGFR3 serves as a novel tau receptor to mediate the Aβ-induced tau internalization that results in the Aβ–tau synergy observed in AD.

### Internalized tau ruptures endocytic vesicles

Pathological amyloid proteins undergo vesicle rupture following endocytosis during cell-to-cell propagation in neurodegenerative diseases^45^, which led us to investigate the cytoplasmic events after Aβ-induced tau uptake. We first used a bimolecular fluorescence complementation assay, which enables visualization of protein‒protein interactions between spatially disparate proteins, to quantify the interaction between extracellular tau (VN-hTau) and cytoplasmic tau (VC-hTau) (Supplementary Fig. 6a). We found that Aβ pretreatment induced significantly more Venus signals, indicating that Aβ-induced tau uptake led to more endocytic vesicle rupture containing extracellular VN-hTau following tau internalization (Supplementary Fig. 6b–d). Additionally, we used a galectin-3 binding assay to visualize vesicle rupture following Aβ-induced tau uptake. Galectin-3 binds to accessible β-galactoside sugars on ruptured endocytic vesicles^46^. We observed that the Aβ-pretreated group was more likely to exhibit condensed punctate structures of galectin-3-mCherry (Supplementary Fig. 6e, f). Taken together, our data suggest that Aβ dependently internalized extracellular tau is released into the cytosol as endocytic vesicles rupture, where it interacts with cytosolic tau in neurons.

### Increased FGF2 in AD accelerates tau uptake in neurons

**Fig. 4:**
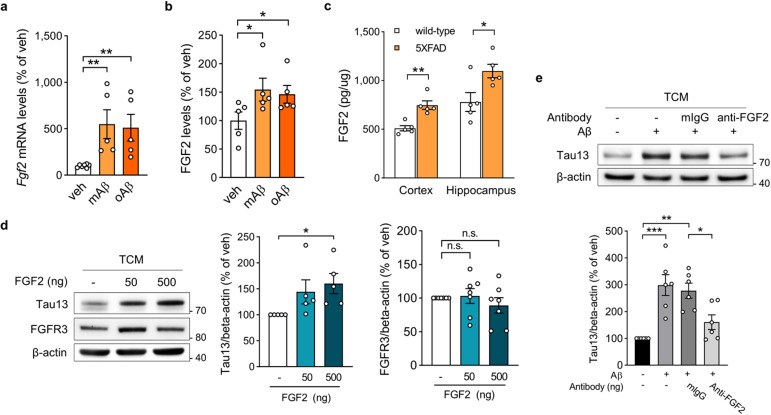
FGF2 secretion induced by Aβ accelerates FGFR3-mediated tau uptake. **a** *Fgf2* mRNA levels in HT22 cells after monomeric or oligomeric Aβ treatment for 16 h. One-way ANOVA with Tukey’s test. **b** Secreted FGF2 levels in cell medium after monomeric or oligomeric Aβ treatment for 16 h, as measured by ELISA. One-way ANOVA with Tukey’s test. **c** FGF2 levels in the cortex and hippocampus of 11-month-old wild-type and 5XFAD mice were measured by ELISA. Unpaired two-tailed t test. **d** Representative immunoblots and quantification of human tau and FGFR3 in HT22 cells after incubation with FGF2 and TCM for 24 h. Human tau and FGFR3 signals were normalized to those of β-actin. One-way ANOVA with Tukey’s test. **e** Representative immunoblots and quantification of human tau in HT22 cells after incubation with neutralizing antibodies against secreted FGF2 and TCM. Human tau signals were normalized to those of β-actin. One-way ANOVA with Tukey’s test. The data are presented as the mean ± SEM. *P < 0.05, **P < 0.01, ***P < 0.001, and ****P < 0.0001.

FGF secretion from damaged tissues leads to the activation of FGFR3 signaling, which has multiple functions in regeneration and wound healing^43,47^. Among the 22 FGF family members, the most common is FGF-2 (basic FGF), which is highly expressed in the brain. FGF2 is elevated in AD patient brains and injured brains^48,49^. Neurons damaged by oligomeric Aβ or glutamate toxicity secrete FGF2 as a neuroprotective response^50^. As FGF2 is a ligand for FGFR3, we investigated whether Aβ increases FGFR3-mediated tau uptake by inducing FGF2 secretion from recipient cells. We found that *Fgf2* mRNA and secreted FGF2 levels were significantly increased in recipient HT22 cells after monomeric or oligomeric Aβ treatment (Fig. 4a, b). We next measured FGF2 levels in 5XFAD mice, which develop severe amyloid pathology^21^. In both cortical and hippocampal tissues, 5XFAD mice expressed higher FGF2 levels than did age-matched wild-type mice (Fig. 4c). Unlike changes in FGF2, FGFR3 levels were not altered by Aβ treatment or amyloid pathology (Supplementary Fig. 7). To assess the impact of tau pathology on FGF2 expression, we analyzed FGF2 expression in human mutant tau (P301L)-overexpressing hippocampal neurons (Supplementary Fig. 8a) and tauopathy model mice (Supplementary Fig. 8b, c). Tau pathology had no effect on FGF2 expression, but *Fgf2* expression was mildly elevated in the hippocampus of tauopathy model mice. These results indicate that, compared to tau pathology, amyloid pathology exerts more potent effects on FGF2 secretion in the brain. To determine whether additional FGF2 treatment accelerates tau uptake, we transferred mouse FGF2-containing TCM to HT22 cells. Increasing concentrations of FGF2 in TCM increased tau uptake without affecting FGFR3 levels (Fig. 4d). In addition, the antibody-mediated blockade of FGF2 in TCM inhibited the ability of Aβ to accelerate tau uptake (Fig. 4e). Taken together, our findings suggest that the FGF2 secretion induced by amyloid pathology is sufficient to promote FGFR3-mediated tau uptake in neurons.

### Neuronal FGFR3 deficiency suppresses tau uptake, pathology, and cognitive deficits in AD model mice

**Fig. 5:**
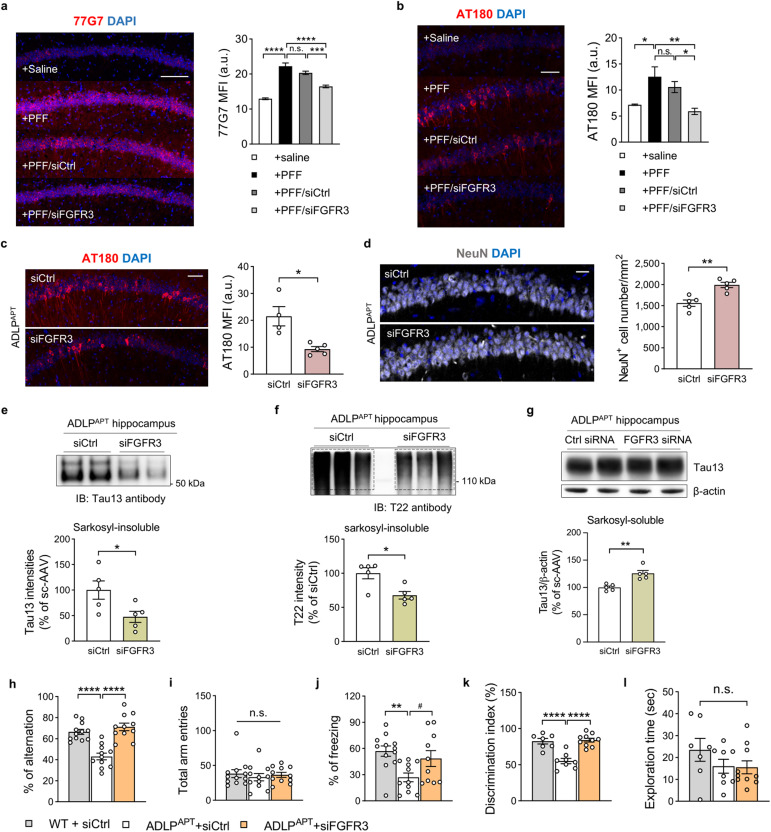
FGFR3 mediates tau uptake and tau-related pathology in AD model mice. Representative image and quantification of the microtubule-binding domain of tau (**a**, 77G7) and phosphorylated tau (**b**, AT180) after the injection of preformed tau fibrils (PFFs), siCtrl or siFGFR3 virus into ADLP^Tau^ mice. One-way ANOVA with Tukey’s test. **c** Representative image and quantification of phosphorylated tau (AT180) in the CA1 region of ADLP^APT^ mice injected with siCtrl or siFGFR3 virus. Scale bar, 50 μm. Unpaired two-tailed t test. **d** Representative image and neuronal cell number in the CA1 region of ADLP^APT^ mice injected with siCtrl or siFGFR3 virus. Scale bar, 20 μm. Unpaired two-tailed t test. Representative immunoblots and quantification of human tau (**e**) and oligomeric tau (**f**) in sarkosyl-insoluble and sarkosyl-soluble (**g**) fractions from the ipsilateral hippocampus of ADLP^APT^ mice after injection of siCtrl or siFGFR3 virus. Unpaired two-tailed t test. The percentage of alternations (**h**) and total arm entries (**i**) in the Y-maze test. One-way ANOVA with Tukey’s test. **j** The percentage of freezing in the CFC test. One-way ANOVA with Tukey’s test. The discrimination index (**k**) and exploration time (**l**) in the novel object recognition task. One-way ANOVA with Tukey’s test. The data are presented as the mean ± SEM. ^#^*P* < 0.05 (unpaired t test), *P < 0.05, **P < 0.01, ***P < 0.001, and ****P < 0.0001.

To examine the effect of FGFR3 deficiency on tau uptake and tau-related pathology in vivo, we knocked down FGFR3 expression using an adeno-associated virus 9 (AA9) encoding FGFR3-targeting or scrambled siRNA, which primarily transduces neuronal populations rather than other cell types (Supplementary Fig. 9a, b)^51^. FGFR3 knockdown led to a 53% reduction in FGFR3 expression in neurons (Supplementary Fig. 9c). To determine whether FGFR3 is required for the uptake of AD-associated pathogenic tau in neurons, we exogenously injected preformed synthetic human tau fibrils (PFFs) with AAV9-siFGFR3 into tauopathy model mice, which are known to be internalized and initiate tauopathy with tau propagation^52^. Interestingly, we observed that FGFR3 knockdown significantly inhibited exogenous PFF uptake and decreased tau phosphorylation in neurons 4 weeks later, as assessed by immunostaining with a 77G7 antibody targeting the microtubule-binding domain of tau and an AT180 antibody (Fig. 5a, b). These data indicate that FGFR3 is required for extracellular tau uptake in neurons.

Next, we examined the effect of FGFR3 deficiency on tau-related pathology in ADLP^APT^ mice, which develop severe amyloid and tau pathologies in the brain^6^. We found that phosphorylated tau levels (AT180) were decreased in FGFR3 knockdown ADLP^APT^ mice at 12 weeks postinjection (Fig. 5c). Additionally, we detected an increase in the number of neuronal cells in the CA1 region (Fig. 5d), indicating that decreased tau pathology restores neuronal density.

To quantify the tau aggregates in the hippocampus, we isolated them by sarkosyl fractionation. We first observed a significant reduction in both monomeric human tau and oligomeric human tau levels in the sarkosyl-insoluble fraction isolated from FGFR3-knockdown ADLP^APT^ mice compared with those in the control (Fig. 5e, f). Interestingly, unlike the impact of FGFR3 deficiency on sarkosyl-insoluble tau aggregates, FGFR3 deficiency led to increased monomeric human tau levels in the sarkosyl-soluble fraction (Fig. 5g). Taken together, these data suggest that FGFR3 contributes to tau aggregation by mediating extracellular tau uptake in neurons.

Reducing tau pathology, which is strongly correlated with cognitive dysfunction in AD, alleviated memory loss in AD model mice^53,54^. We observed that FGFR3 knockdown in ADLP^APT^ mice restored cognitive deficits in three different behavioral tests, consistent with prior studies (Fig. 5h–l). We first used the Y-maze to assess spatial working memory. Compared with wild-type mice, FGFR3 knockdown ADLP^APT^ mice had intact spatial memory, which rescued their alternation percentage (Fig. 5h). There was no between-group difference in the total number of entries (Fig. 5i). To examine long-term memory reconsolidation, we quantified contextual fear memory in the contextual fear conditioning test. The freezing rate of FGFR3-knockdown ADLP^APT^ mice was restored, whereas that of control mice was not (Fig. 5j). Additionally, in the novel object recognition test, FGFR3 knockdown ADLP^APT^ mice discriminated the novel object at similar levels as wild-type mice (Fig. 5k, l). FGFR3 knockdown also rescued the spatial memory deficits of ADLP^Tau^ mice in the Y-maze test, as indicated by reduced phosphorylated tau levels (Supplementary Fig. 10). Taken together, these data show that downregulating neuronal FGFR3 decreases tau uptake and neurotoxic tau pathology, thereby preventing neuronal cell death and restoring cognitive impairments in AD model mice.

## Discussion

Over the last decade, researchers have developed the amyloid cascade hypothesis, which proposes that amyloid pathology acts as an initiator for subsequent tau pathology and neurodegeneration in AD^55^. However, the therapeutic targeting of Aβ alone or tau alone has failed to alleviate the clinical symptoms of AD or impede its progression^56–58^. The insufficiency of sole anti-tau or anti-Aβ therapy indicates that we must elucidate the complex interrelationship of Aβ–tau. Accumulating evidence suggests that Aβ and tau act independently without direct interaction but reciprocally cooperate to exert synergistic effects on AD pathogenesis^16,59^. Amyloid plaque deposition and tau aggregation are initiated in different brain regions. Plaques are first deposited in the neocortex region, whereas NFT formation progresses from the limbic and entorhinal cortices to the hippocampus^60,61^. As the disease progresses, the spread of tau aggregates is closely related to the deposition of plaques and is synergistic with cortical plaques^62^. However, the molecular mechanisms underlying the synergy between Aβ and tau remain poorly understood. Here, we confirm the hypothesis that Aβ and tau act independently but synergistically to accelerate tau uptake, further contributing to tau pathology in neurons.

Soluble Aβ can affect various aspects of tau pathology, including its secretion, aggregation, and internalization. Soluble Aβ induces neuronal hyperactivity and network abnormalities that are reflected by seizure-like symptoms in AD^63^. Since the release of tau can be modulated pharmaceutically or electrophysiologically by neuronal activity^41,64^, it is presumed that Aβ-induced hyperexcitability stimulates the transsynaptic spread of tau. However, we herein observed that Aβ-treated neurons do not secrete more tau than nontreated neurons. This finding excludes the possibility that Aβ drives tau secretion in neuronal cell lines and neurons. Tau may exist in the extracellular environment, and tau aggregates may thus be exposed due to neuronal death caused by Aβ toxicity; however, this does not explain the propagation of tau in the early and middle stages of AD. Most of the existing studies have focused on the ability of Aβ to enhance tau aggregate formation by altering posttranslational modification and cross-seeding^7,11,65^. Although evidence from various AD animal models in which Aβ and tau are coexpressed supports the idea that Aβ promotes tau aggregation and propagation as the disease progresses, it is not clear whether Aβ induces tau aggregation and/or propagation in neurons.

Despite the well-known significance of pathological tau propagation in neurodegenerative diseases^66,67^, few studies have identified molecular factors that may mediate the spread of tau among neurons. We herein hypothesized that Aβ stimulates the spread of tau, specifically its internalization, leading to further tau pathology and propagation. A prior study suggested the ability of Aβ to promote tau uptake, but the underlying molecular mechanism was not addressed in detail^15^. Low-density lipoprotein receptor-related protein 1 (LRP1) has recently been suggested to be a common factor contributing to the co-occurrence of amyloid and tau pathologies. However, LRP1 appears to act as a receptor involved in the internalization of Aβ or tau rather than as a mediator of the Aβ–tau axis^42^. Another receptor, cellular prion protein (PrP^C^), contributes to the interplay between Aβ and tau and is a prerequisite for tau pathologies in AD^68,69^. However, the binding of PrP^c^ to Aβ causes tau aggregation by regulating the posttranslational modification of tau and is thus insufficient to explain its cell-to-cell propagation. Neither receptor is sufficient to explain the effect of Aβ on tau internalization, and the detailed mechanisms of the Aβ–tau synergy have yet to be explained.

We demonstrate that the internalization of a novel tau receptor, FGFR3, is regulated by Aβ and plays a crucial role in neuronal tau uptake. Proteomic analysis reveals that FGFR3 is enriched in endocytic vesicles under Aβ-stimulated conditions, where it directly interacts with extracellular tau. In the Aβ–FGFR3–tau axis, the internalization of tau by FGFR3 is not directly promoted by the physical interaction of Aβ. When neurons are degenerated by oligomeric Aβ or glutamate toxicity, released FGF2 exerts neuroprotective effects on neurons or glial cells expressing FGFR^50^. The members of the FGF family are secreted from different cell types for tissue repair and regeneration in damaged tissues^70^. Another prior study showed therapeutic effects of systemic FGF2 administration, resulting in reduced amyloid pathology^71^. However, we observed that secreted FGF2 triggered by Aβ paradoxically accelerates tau uptake and exacerbates tau pathology despite its role as a neuroprotective growth factor.

Although we focused on the role of neuronal FGF2–FGFR3 signaling in tau internalization, we note that other FGFR3 ligands and/or different cell types in the brain might also contribute to Aβ-induced FGFR3/tau internalization. We herein show that FGF2, a representative FGF family member in the brain, participates in tau internalization; however, other FGFR3 ligands, such as FGF1 and/or FGF9, may also affect tau uptake^72^. In AD, reactive astrocytes also strongly express and secrete excess FGF1 (an acidic FGF)^73,74^, which is another ligand for FGFR3. Defining additional factors involved in FGFR3-mediated tau uptake is important for future studies. As FGFR3 is known to form a heterocomplex with HSPG and FGF^75,76^, we speculate that HSPG may increase the ability of FGFR3 to internalize tau. In addition, glycosaminoglycans (GAGs), including the heparan sulfate of HSPG, have altered the ability to bind FGF and tau in AD patients^77^, suggesting that the HSPG–FGFR3 heterocomplex may synergistically interact with tau in the presence of Aβ.

Finally, FGFR3-mediated tau uptake significantly accelerates tau-related AD pathologies, including NFT formation and cognitive dysfunction. Neuronal *Fgfr3* knockdown inhibits tau uptake and aggregation, leading to the attenuation of memory loss. Notably, neuronal *Fgfr3* knockdown led to a significant decrease in tau aggregates isolated from the sarkosyl-insoluble fraction but an increase in human tau levels in the sarkosyl-soluble fraction. These results indicate that neuronal FGFR3 acts as an upstream regulator of tau-related AD pathology and is implicated in tau aggregation by contributing to tau internalization. Consistent with previous reports that insoluble tau aggregates are strongly implicated in cognitive decline in AD^78^, these data provide therapeutic insight that the disturbance of the dynamic equilibrium of tau aggregation by neuronal FGFR3 deficiency could effectively reduce and delay the progression of tau-related AD pathologies.

**Fig. 6:**
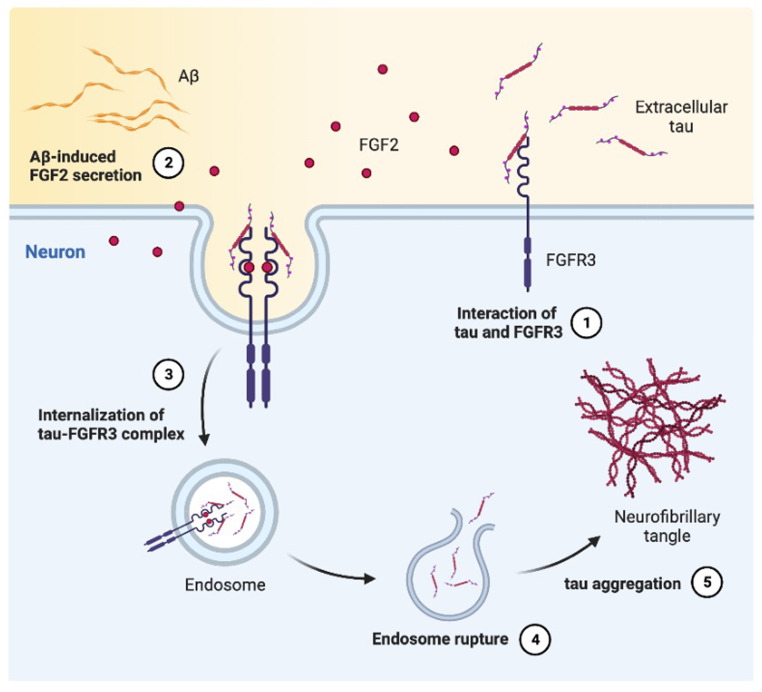
Schematic of Aβ-induced tau uptake mediated by FGFR3. FGFR3 acts as a surface receptor for tau uptake, and its internalization is accelerated by Aβ-induced FGF2 secretion. In the brains of AD model mice, activation of the FGF2/FGFR3 pathway by Aβ exacerbates tau internalization in neurons, leading to tau propagation and NFT formation.

Our study highlights that Aβ induces tau uptake via FGFR3 in AD. Based on the amyloid cascade hypothesis, Aβ critically triggers the secretion of FGF2 from neurons, leading to the internalization of tau-bound FGFR3 as a novel mediator of tau uptake (Fig. 6). Our finding that FGF2–FGFR3 signaling lies at the hub of tau propagation improves our understanding of the accelerated tau spread observed in AD patients with both amyloid and tau pathologies.

### Supplementary information

Supplementary information

Supplementary Table 1
