# Supplementary material for: FGFR3 drives Aβ-induced tau uptake
Source: Exp Mol Med. 2024 Jul 1;56(7):1631–42. doi: 10.1038/s12276-024-01274-3 (PMC11297141; doi:10.1038/s12276-024-01274-3)
Supplement: Supplementary file 1 — Supplementary information [file 12276_2024_1274_MOESM1_ESM.pdf]

Supplementary Materials

FGFR3 drives Aβ-induced tau uptake

Dong Kyu Kim[1,2,†], Kyujin Suh[1,2,†], Junho Park[3], Sang-Eun Lee[1,4], Jihui Han[1,2], Sunghoe Chang[1,4], Young Soo Kim[1], Inhee Mook-Jung[1,2*]

Supplementary Material and Methods

Supplementary Figures – Supplementary Fig. 1-10

Supplementary Material and Methods

Quantitative real-time PCR (qPCR)

Total RNA was isolated using a RNeasy Plus Mini Kit (QIAGEN). According to the manufacturer's protocols, cDNA was synthesized from 100 ng total RNA using a Maxime RT PreMix kit (iNtRON Biotechnology). Quantitative real-time PCR was performed with the KAPA SYBR FAST mix (Applied Biosystems). The expression levels of *Fgf2* and *Fgfr3* were analyzed by the comparative Ct method and normalized to the expression level of *Rps18* (18s rRNA). The utilized primer sequences were as follows: *Fgfr3* forward 5'-CCTGTGTAGTTGAGAACAAGTTT-3'; *Fgfr3* reverse 5'-GTGTTGGAGTTCATAGAGGAGT-3'; *Fgf2* forward 5'-AGCGGCTCTACTGCAAGAAC-3'; *Fgf2* reverse 5'-AGCAGACATTGGAAGCAGT-3'; *Rps18* forward 5'-GTAACCCGTTGAACCCCATT-3'; *Rps18* reverse 5'-CCATCCAATCGGTAGTAGCG-3'.

Co-immunoprecipitation

Cells were lysed in 1% Triton X-100 in TBS buffer (50 mM Tris HCl, 150 mM NaCl, pH 7.4) containing a protease inhibitor, a phosphatase inhibitor cocktail, and phenyl-methylsulfonyl fluoride (PMSF) (Sigma-Aldrich). For co-immunoprecipitation of FGFR3 and tau or Aβ, antibodies against Tau-13 (Abcam), FLAG (Sigma), HA (Santa-cruz), or $A\beta_{17\text{-}24}$ (Abcam) were crosslinked to protein A/G agarose beads (Santa Cruz) by BS3 (Thermo Fisher Scientific) according to the manufacturer's instructions, and then incubated with lysates overnight at 4°C. The next day, precipitates were heated at 95°C for 5 min in SDS-PAGE sample buffer, resolved by SDS-PAGE, and subjected to Western blot analysis.

ELISA

Sandwich ELISA kits for mouse FGF2 (MFB00, R&D Systems) and human tau (khb0041, Thermo Fisher Scientific) were used to quantify FGF and human tau, respectively, in brain tissue and cell culture supernatants according to the manufacturer's instructions.

TCA precipitation

To examine secreted proteins, TCA (final concentration, 10%) was used to precipitate proteins from cell-conditioned medium. Since fetal bovine serum would interfere with this precipitation, reduced-serum Opti-MEM was used for these experiments. Samples were vortexed and incubated overnight on a rotator at 4°C. After centrifugation at 13,000 rpm for 30 min at 4°C, the obtained protein pellets were washed twice with ice-cold acetone, air dried, and dissolved in 2x sample buffer for Western blot analysis.

DNA constructs and transfection

For making the tau-conditioned medium, pRK5-EGFP-Tau and pRK5-EGFP-TauP301L were used and supplied by Addgene. For investigating FGFR3 cytosolic domain effects, pSH1/M-FGFR3-Fv-Fvls-E was used and supplied by Addgene (Plasmid #15287). For FGFR3 gain of function experiments, pCMV6-FGFR3-Myc-DDK was used and supplied by Origene. For the galectin-3 binding assay, pmCherry-Gal3 was used and supplied by Addgene (Plasmid #85662). For transfection into HT22 cells, DNA constructs were pre-mixed with Lipofectamine LTX (Invitrogen) and applied to cells in Opti-MEM medium (Gibco).

Cell culture and tau-conditioned medium

HT22 cells were cultured in Dulbecco's modified Eagle's medium (DMEM, Hyclone) supplemented with 10% fetal bovine serum (Hyclone) and 1% penicillin/streptomycin (Sigma-Aldrich) at 37°C in a 5% CO_2 incubator. Tau-conditioned medium (TCM) was collected from HT22 cells overexpressing GFP-hTauP301L or GFP-hTauWT(wild-type). Each conditioned medium was centrifuged at 2,000 g for 10 min to remove cell debris and/or dead cells. TCM was freshly produced each time without any special concentration process.

Supplementary Figures

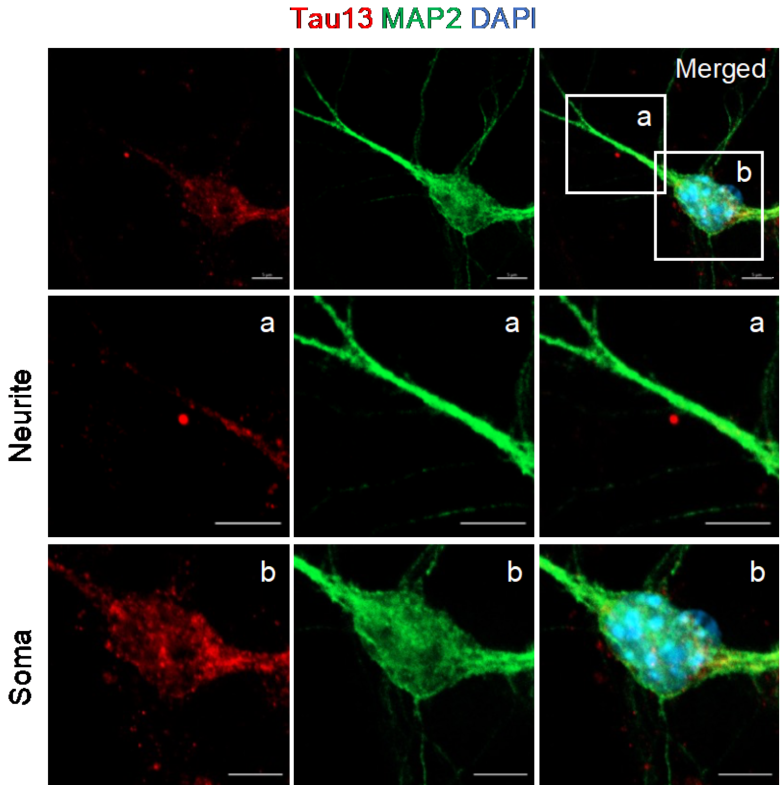

Supplementary Fig. 1: Cellular distribution of internalized tau in primary neurons.

Primary neurons showing internalized tau (Tau13) in the neurite (a) and soma regions (b). Scale bar, 5 μm.

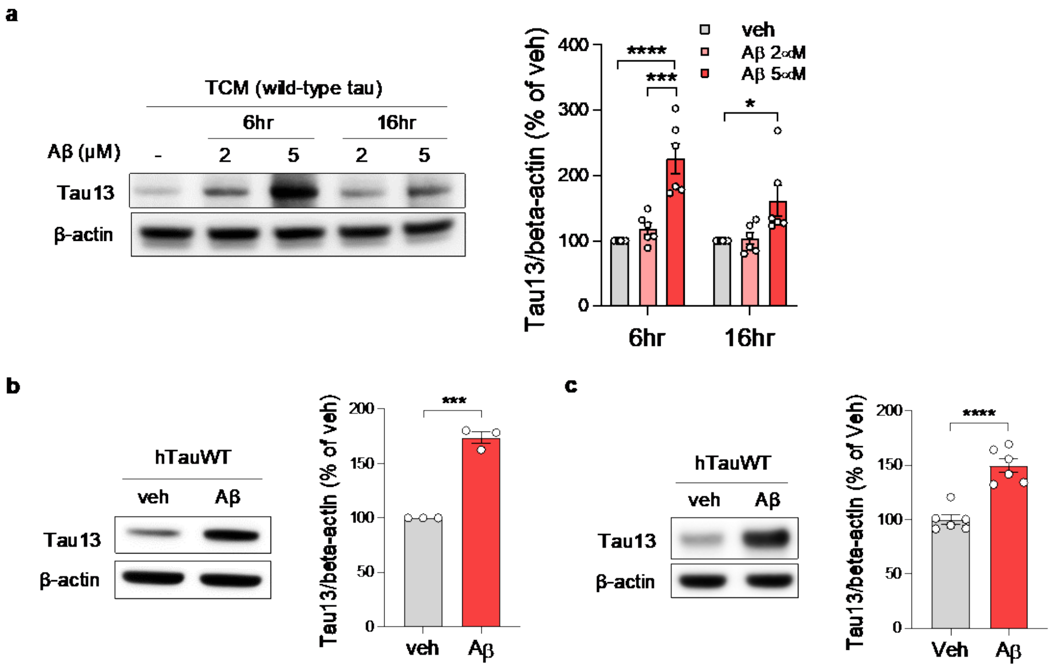

Supplementary Fig. 2: Aβ accelerates uptake of extracellular wild-type human tau.

a Representative immunoblots and quantification of human tau in HT22 cells incubated with TCM (wild-type human tau) after Aβ treatment. Human tau signals were normalized with β-actin. Human tau signals were normalized with β-actin. Two-way ANOVA (n = 6). **b, c** Representative immunoblots and quantification of human tau in HT22 cells (**b**) and primary neurons (**c**) incubated with human wild-type tau after Aβ treatment. Unpaired two-tailed t-test (n = 3 (**b**), 6 (**c**)). Data are presented as mean ± SEM. *P < 0.05, **P < 0.01, ***P < 0.001, and ****P < 0.0001.

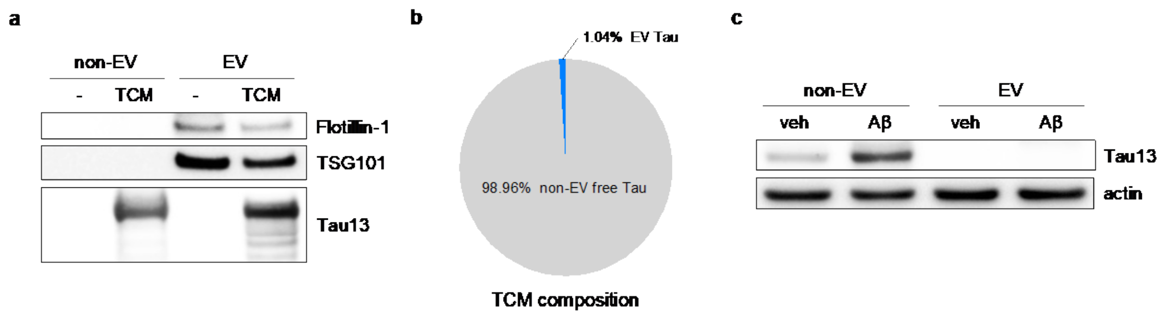

Supplementary Fig. 3: Aβ accelerates the internalization of free-form of human tau, which is a major form of secreted human tau in TCM.

a Extracellular vesicles (EV) were isolated from tauP301L-conditioned medium (TCM). Secreted human tau was detected in both TCM without EV (non-EV) and EV assessed by Western blot analysis with exosome markers, flotilin-1 and TSG101. **b** 98.96% of secreted tau in TCM exists in the free form, which is measured by human tau ELISA. **c** Representative immunoblots of human tau in HT22 cells incubated with either non-EV or EV following Aβ (5 μM) treatment.

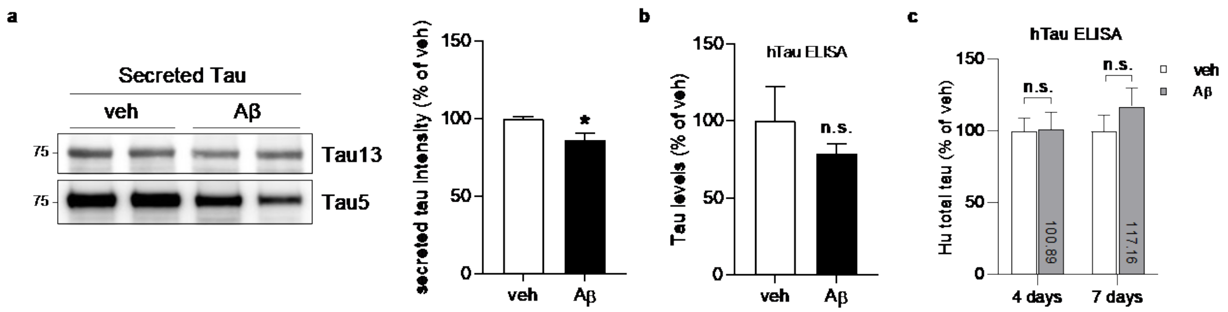

Supplementary Fig. 4: Secretion of tau is not induced by Aβ.

a Representative immunoblots and quantification of secreted human tau in tau-conditioned medium (TCM), which is precipitated by TCA precipitation. Unpaired two-tailed t-test (n = 4). **b** Secreted tau in TCM were analyzed by human tau ELISA. Unpaired two-tailed t-test (n = 6). **c** Primary cortical rat neurons were treated with Aβ 1 µM for 4 (14 days in vitro (d.i.v.)) or 7 days (17 d.i.v.). Neuronal conditioned medium was obtained and used for the quantification of secreted human tau by ELISA. Unpaired two-tailed t-test (n = 4). Data are presented as mean ± SEM. *P < 0.05, **P < 0.01, ***P < 0.001, and ****P < 0.0001.

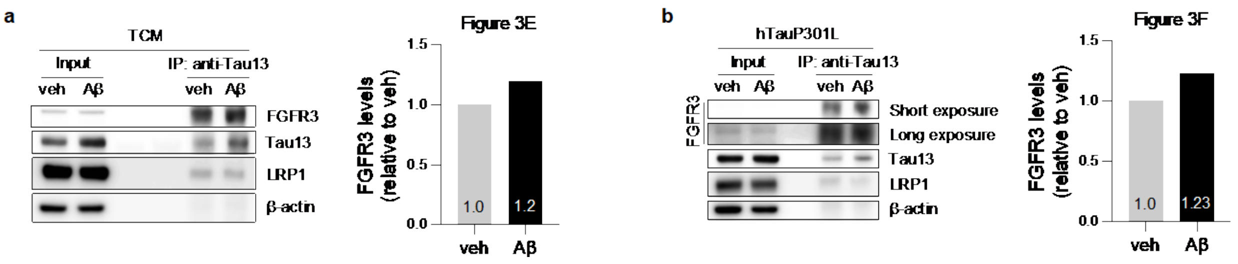

Supplementary Fig. 5: Quantification of the relative change in the amount of FGFR3 binding to extracellular tau in primary neurons for Figure 3E and F.

a, **b** Immunoblots and quantification of coimmunoprecipitation of human tau in HT22 cells (**a**) and primary neurons (**b**).

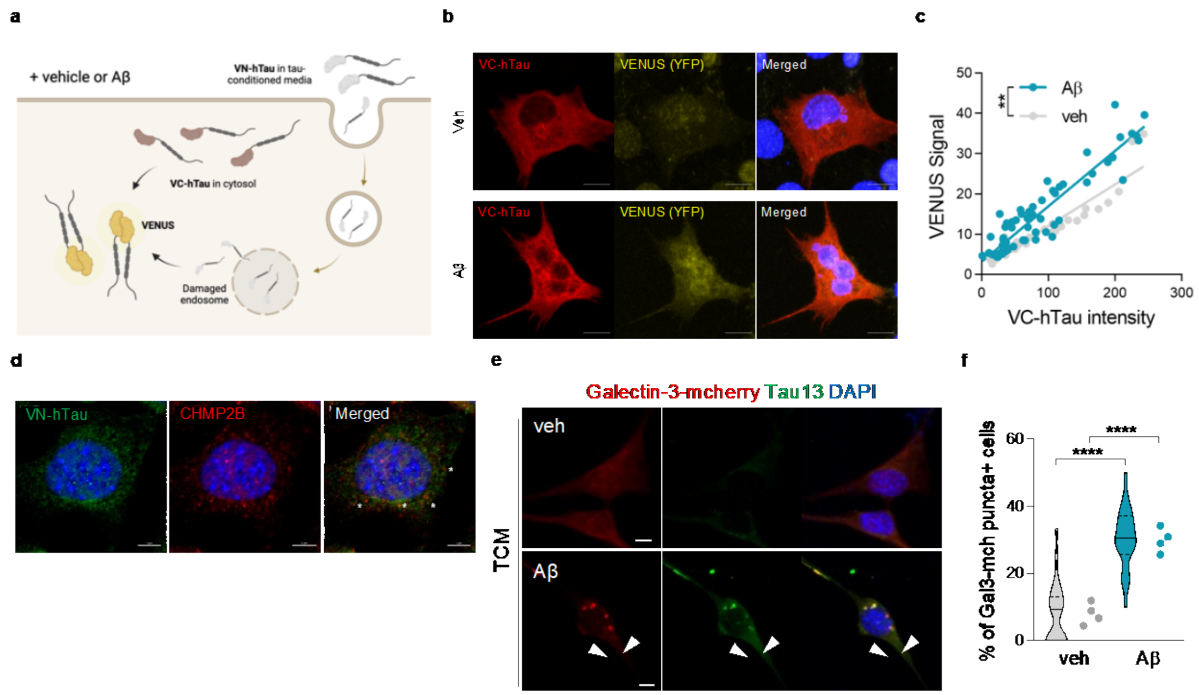

Supplementary Fig. 6: Aβ-induced tau uptake ruptures endocytic vesicles.

a Overview schematic of the BiFC assay. **b** Representative images of VENUS signals in HT22 cells overexpressing VC-hTau, followed by Aβ pretreatment and VN-hTau-conditioned medium incubation. **c** Correlation analysis between VENUS signals and VC-hTau signals in the vehicle or Aβ group. Linear regression analysis (*n* = 38-64 cells/group). Scale bar, 10 μm. **d** Recipient cells showing co-localization between VN-hTau and CHMP2B. Scale bar, 5 μm. **e** Representative images of the galectin-3-mcherry assay in HT22 cells overexpressing galectin-3-mcherry, followed by Aβ pretreatment and TCM incubation. Arrow heads indicate co-localization of galectin-3-mcherry and human tau. **f** The percentage of galectin-3-mcherry^{+} cell number. Unpaired t-test (*n* = 177, 164 cells/each group, 4 replicates/group). Data are presented as mean ± SEM. $^{**}P < 0.01$ and $^{****}P < 0.0001$.

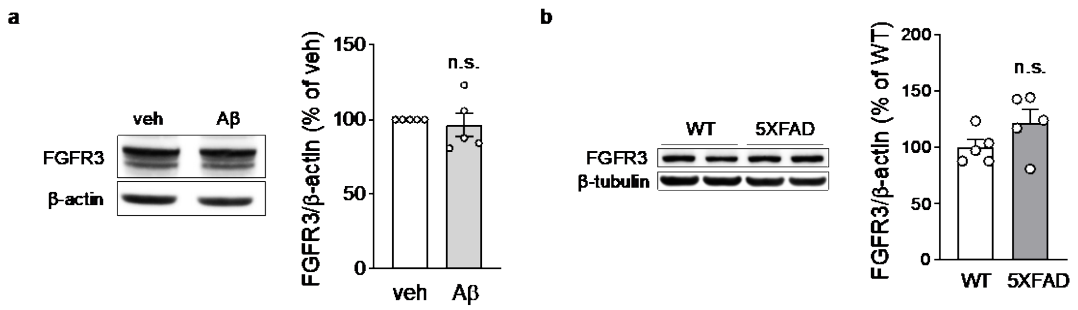

Supplementary Fig. 7: Aβ does not change FGFR3 levels.

a Representative immunoblots and quantification of FGFR3 in HT22 cells after Aβ treatment (5 μM, 16 hrs). FGFR3 signals were normalized to β-actin. Unpaired two-tailed t-test (*n* = 5). **b** Representative immunoblots and quantification of FGFR3 in the hippocampus of 11-month-old 5XFAD and wild-type mice. FGFR3 signals were normalized to β-actin. Unpaired two-tailed t-test (*n* = 5 mice/group). Data are presented as mean ± SEM.

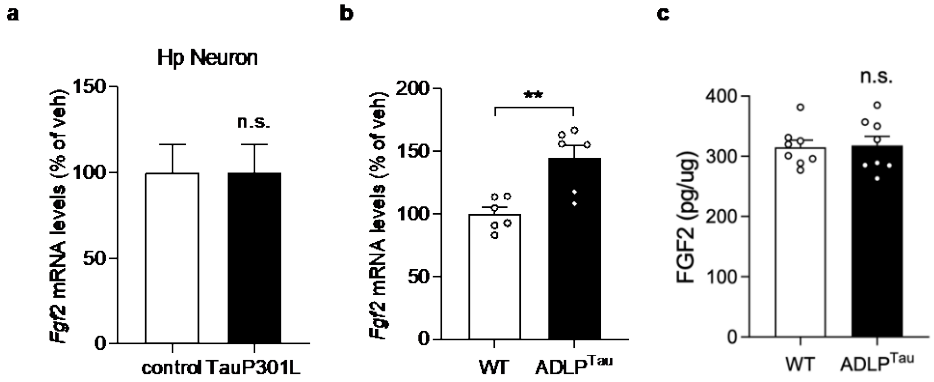

Supplementary Fig. 8: Human tau overexpression mildly affects *Fgf2* expression.

a *Fgf2* mRNA levels in mouse primary hippocampal neurons (14 d.i.v) expressing either control vector or human mutant tuaP301L. Unpaired two-tailed t-test (n = 7). **b** *Fgf2* mRNA levels in the hippocampus of 15-month-old ADLP^{Tau} and wild-type mice. Unpaired two-tailed t-test (n = 6 mice/group). **c** FGF2 amounts measured by ELISA in the hippocampus of 15-month-old wild-type and ADLP^{Tau} mice (n = 8 mice/group). Unpaired two-tailed t-test. Data are presented as mean ± SEM. **P < 0.01.

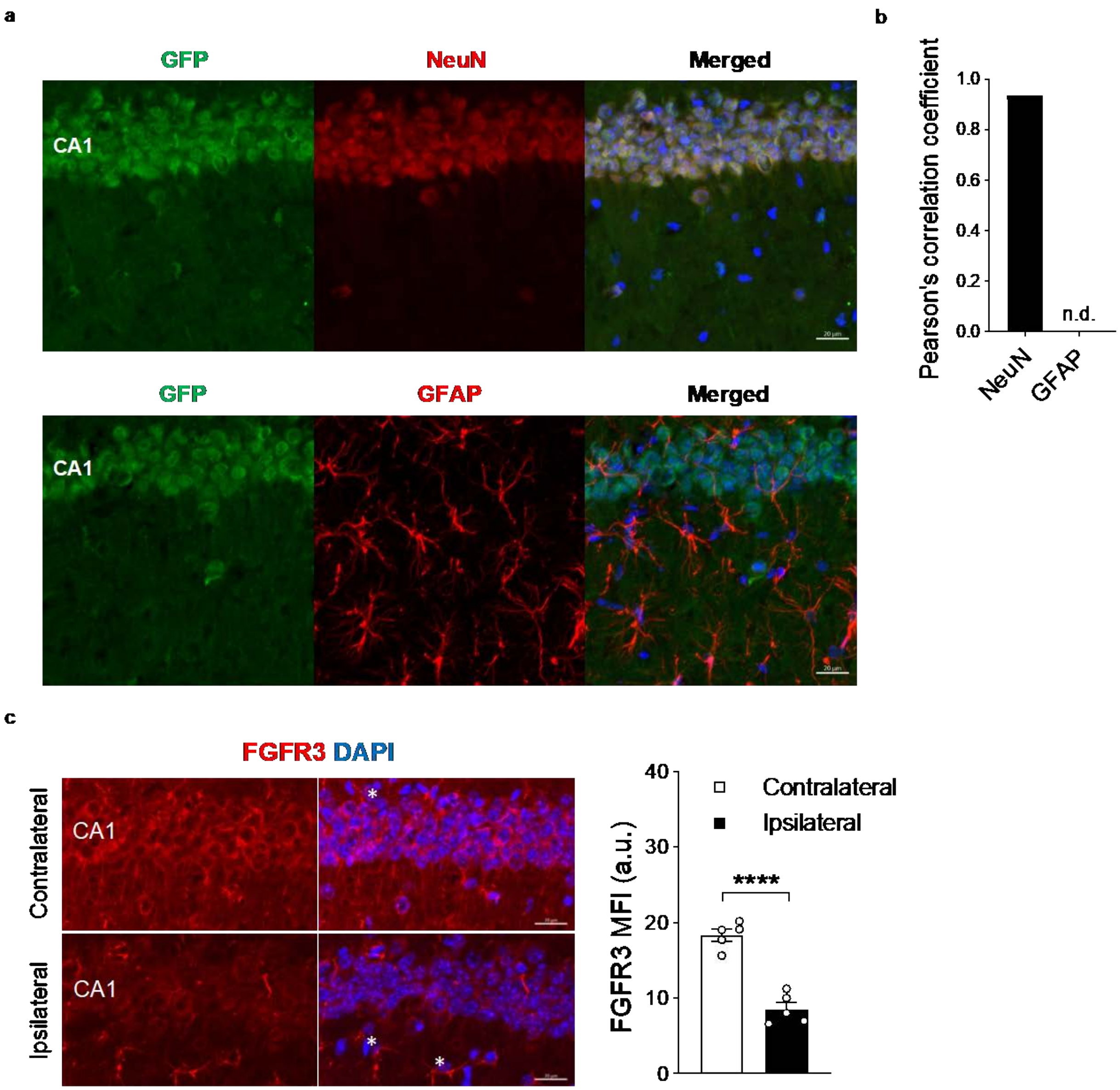

Supplementary Fig. 9: Characterization of AAV-siRNA system for gene knockdown.

a Immunostaining of transduced cells labeled with AAV-siCtrl-GFP and cell-type markers (NeuN or GFAP). Scale bar, 20 µm. **b** Pearson's correlation coefficient of co-localization. N.d. stands for not detected value. **c** Representative images and quantification of FGFR3 in the CA1 of FGFR3 knockdown ADLP^{APT} mice. Asterisks indicate astrocytes expressing FGFR3. Scale bar, 20 µm. Unpaired two-tailed t-test ($n = 5$ mice/group). Data are presented as mean ± SEM. ****$P < 0.0001$.

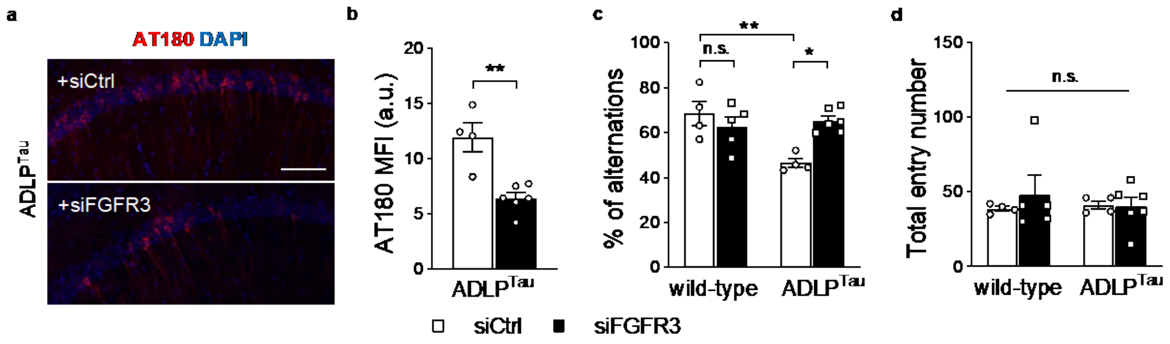

Supplementary Fig. 10: FGFR3 knockdown alleviates tau pathology and memory impairment in tauopathy model mice.

a, b Representative images (**a**) and quantification (**b**) of phosphorylated human tau (Thr231, AT180) after injection of siCtrl or siFGFR3 virus into ADLP^{Tau} mice. Unpaired two-tailed t-test (n = 4-6 mice/group). **c, d** The percentage of alternations (**c**) and total entry number (**d**) in the Y-maze test. Two-way ANOVA (n = 4-6 mice/group). Data are presented as mean ± SEM. *$P < 0.05$, **$P < 0.01$.
